# Health-Related Factors Associated with Discrepancies between Children’s Potential and Attained Secondary School Level: A Longitudinal Study

**DOI:** 10.1371/journal.pone.0168110

**Published:** 2016-12-22

**Authors:** Iris van der Heide, Ulrike Gehring, Gerard H. Koppelman, Alet H. Wijga

**Affiliations:** 1 Centre for Nutrition, Prevention and health Services, National Institute for Public Health and the Environment, BA Bilthoven, Netherlands; 2 Division Environmental Epidemiology, Institute for Risk Assessment Sciences, Utrecht University, Utrecht, Netherlands; 3 Department of Pediatric Pulmonology and Pediatric Allergology, University of Groningen, University Medical Center Groningen, Beatrix Children's Hospital, GRIAC Research Institute, Groningen, Netherlands; University Children's Hospital Tuebingen, GERMANY

## Abstract

**Objectives:**

This longitudinal study examines children’s health-related characteristics in relation to discrepancies between their educational potential assessed by a cognitive test in primary school at age 11 and their attained secondary school level at age 14.

**Methods:**

Data were used from 1510 participants of the Dutch PIAMA (Prevention and Incidence of Asthma and Mite Allergy) birth cohort. Multiple multinomial logistic regression analyses were used to estimate the associations between indicators of physical health, psychosocial health, lifestyle, sleeping patterns and stressful life events and attending a higher or lower level of secondary education than indicated by the cognitive test.

**Results:**

We found no evidence that physical health contributes to discrepancies between the potential and attained level of secondary education, but attention disorders and stressful life events (nasty experiences and parents’ divorce) were associated with educational attainment below children’s potential level. Furthermore, substance use (alcohol, drugs and smoking) were strongly associated with attending a lower level of secondary education than expected (odds ratios from 2.2 (CI: 1.5–3.3) to 5.0 (CI: 2.8–8.7)).

**Conclusion:**

In this general population study, attention disorders, stressful life events and especially substance use seemed to be more important than physical health for the discrepancy between expected and attained level of secondary education. The longitudinal design of the present study and the broad range of health-related factors that were studied, adds insights to the process of health-selection.

## Introduction

It is well recognized that educational achievement has far-reaching consequences for health later in life [1,2]. In the Netherlands, as in other countries, life expectancy increases with attained level of education [3,4]. It is therefore of great importance, both for their future socio-economic position and for their later health, that children complete the level of education that matches their abilities (their educational potential). It is also important to understand the range of factors that can positively or negatively affect children’s chances to fulfill their educational potential. Parents’ level of education is a well-established and strong determinant of children’s educational achievement (their performance in school or their attended level of education) [5]. In addition, health-related factors, such as lifestyle [6], sleep patterns [7], stress [8,9] and health status [10] can affect educational achievement. A better understanding of the role of health-related factors may facilitate the development of interventions that create a breakthrough in the vicious circle of poorer health status affecting educational achievement affecting health status later in life.

To date, the effect of health on the educational achievement of children is most often studied based on cross-sectional research designs, in which children’s educational achievements are compared to the achievements of their peers. Little is known on health-related factors that influence children’s educational achievement in comparison to their potential as assessed earlier in their school career. In countries with a hierarchical secondary school system, children are directed towards different levels of education at an early age.

In the Netherlands children are directed to a specific level of secondary education based on two criteria. First, scores on a standardized school achievement test, the Central Institute for Test Development (Cito) Test, are used [11]. In most Dutch schools, children have to take this test at the end of their primary school education in order to determine which level of secondary education best suits their abilities. The second criterion is the teachers’ assessment of the child with regards to a suitable secondary school level for him or her. The teacher’s assessment is based on the entire school career, skills and characteristics of the child [12]. If the school uses the Cito-test, the teacher’s assessment mostly corresponds with the secondary school level that is indicated by the Cito-test (65%) [12]. However, when the teacher thinks that the Cito-test score does not represent the child’s usual performance his/her assessment may be that a higher or lower level of secondary education than indicated by the Cito-test score would be the most suitable for the child. Based on the test results and the teacher’s assessment, the child will enter secondary education in one of seven levels ranging from the lowest Dutch secondary educational level that prepares for the labour market to the highest level that prepares for university education. In theory, parents are free to choose the level of secondary schooling they want for their child, but most secondary schools use the Cito-test score and/or the teacher’s school level assessment as an entry requirement, so that in practice the Cito-test score and the teacher’s assessment determine the level at which the child enters secondary education. Both indicators are therefore crucial for the school career of children in the Netherlands. A recent study showed that teacher’s assessment of children’s educational abilities and Cito-test score were only slightly affected by health problems, but more strongly associated with parental education [13].

The aim of our study is to provide insight into the influence of health-related factors on children’s educational career, by comparing children’s attained educational level to their educational potential. Our study builds on the existing body of evidence from cross-sectional studies, indicating associations between health-related factors and educational achievement [6–10]. The following research question will be addressed in this study: Are health-related characteristics associated with discrepancies between the child’s potential, as assessed in primary school, and their attained level of secondary education three years later?

## Methods

### Ethics statement

The Medical Ethical Committees of the participating institutes (Utrecht, age 12 years METC protocol number 07-337/K) approved the study. Parents, carers or guardians gave written informed consent on behalf of all the minors/children involved in the study.

### Data availability statement

The data underlying the findings presented in this paper are available on request. Requests can be submitted to the PIAMA Principal Investigators. Their names and e-mail addresses are listed on the PIAMA website (http://piama.iras.uu.nl/index-en.php#collaboration). The PIAMA data are not freely accessible in the public domain, because this would be in conflict with the agreement between the PIAMA study team and the PIAMA participants. The information participants received at the start of the study (in 1996–1997) included the statement 'the information that we receive from you will only be used for the PIAMA project' and participants gave written informed consent based on this information.

### Study design and population

Data from the Dutch PIAMA (Prevention and Incidence of Asthma and Mite Allergy) study was used. Details of the design of the PIAMA study have been published previously [14]. Pregnant women were recruited from the general population in three different parts of the Netherlands. Their children (n = 3963) were born in 1996/1997 and have been followed from birth onwards. Data on family characteristics, lifestyle and physical as well as psychosocial health were collected by questionnaires in 11 waves of follow-up: at 3 months of age, annually from 1 to 8 years and at 11 and 14 years of age. All parents and caregivers gave written informed consent.

**Fig 1:**
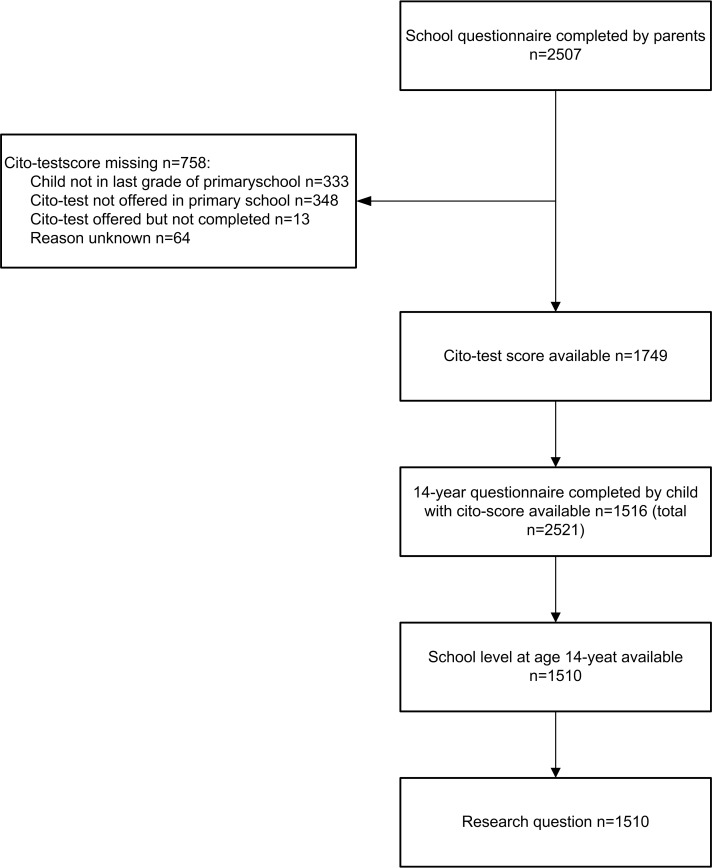
Flow diagram of number of subjects included in the study (baseline n = 3963).

At the age of 11 years, 3541 families (89.4% of baseline population of 3963) were still in the study and questionnaires were completed by the parents (n = 2660, 67% of baseline) as well as the children themselves (n = 2651). If parents and/or the child had completed the 11-years-questionnaire, the parents subsequently received a short additional questionnaire asking about the Cito-test (cognitive test) score and the teacher’s assessment of the child’s educational abilities. These ‘school questionnaires’ were sent to the parents a few weeks after they received the results of the Cito-test and were completed by 2507 parents (63% of baseline). Parents of 1749 children reported the Cito-test score; 694 children had no test score for various reasons (see Fig 1), and for 64 children the reasons for not reporting the Cito-test score is unknown. At the age of 14 years questionnaires were completed by 2521 children (64% of baseline) and 2335 parents (59% of baseline). To answer the research question we included children who completed the 14-year questionnaire and for whom Cito-test scores and teacher’s assessment of their educational abilities were available (n = 1510, 38% of baseline) (see Fig 1).

### Study variables

#### Cognitive test, teacher’s assessment and level of secondary education

Parents reported the child’s Cito-test score and teacher’s assessment, shortly after these became available; the children reported the level of education they were following at the age of 14 years. Cito-test scores range from 501 to 550 and can be translated into levels of secondary education (see S1 Table) [11]. Using the International Standard Classification of Education developed by UNESCO (ISCED 1997), we distinguished 7 different levels of secondary education, as shown in S1 Table, and assigned points to each level. The actual level of secondary education at age 14 years was considered higher or lower than the level indicated by the Cito-test score, if there was a difference of at least 1 point according to the classification presented in S1 Table [12,15]. Repeaters (n = 10) were categorized as performing lower than expected.

#### Socio-demographic characteristics

Since previous research shows that socio-demographic factors are associated with children’s educational achievement, these were taken into account in the current study [16]. Child’s sex, ethnic background and parents’ highest attained level of education were derived from questionnaires completed in the first 2 years of the child’s life.

#### Physical health

All health-related study variables were derived from the 14-years questionnaires. The children reported about their self-perceived general health status (answering the question “How do you think/feel about your health?”, with four answering options ranging from very good to bad). They answered ‘yes’ or ‘no’ to questions on the presence of specific chronic diseases or complaints during the last 12 months i.e. asthma, regular headaches or migraine, and fatigue. The parents reported the number of days the child stayed ill at home during the last 2 months.

#### Psychosocial health

The children reported about their mental health. Mental health was measured by the Mental Health Index (MHI5) and provides a score ranging from 0 to 100 [17,18]. Mental health is considered poor at a score below 61. Parents were asked whether their child was ever diagnosed with one of the following disorders: learning disability (e.g. dyslexia, dyscalculia), attention disorder (e.g. Attention Deficit Disorder (ADD) or Attention Deficit Hyperactivity Disorder (ADHD)), or autistic disorder (e.g. Pervasive Development Disorder–Not Otherwise Specified (PDD-NOS), or Asperger).

#### Lifestyle

The children reported how often they smoked, consumed alcohol and used drugs, answering questions that had a number of frequency categories, including ‘never’, as answering options. Based on the frequency distributions of these variables we created the following dichotomous variables: alcohol consumption in the last 4 weeks (yes/no), smoking (ever versus never) and drug use (ever versus never). Children also reported on TV viewing and computer use (hours per week, added up to a combined variable ‘screen time’) and on the number of school hours skipped in the last 4 weeks.

#### Sleeping patterns

The children reported on the following aspects of sleep: bed times on school days, night time awakenings, difficulty getting up in the morning, feeling rested after waking up on school days and chronotype (being a morning person or evening person).

#### Stressful life events

Children answered the following questions about stress and stressful events: ‘Do you have problems that bother you day and night?’, ‘Have you been bullied during the last 12 months?’ and ‘How many nasty things have you experienced in your life on a scale from 0 (no nasty things) to 10 (a lot of nasty things) since you were 12 years old?’. Parents were asked whether their child had experienced the death of a family member or other person that was close to the child and whether their child had experienced a divorce of his/her parents.

### Statistical analysis

Simple regression analyses and subsequently multinomial logistic regression analyses with and without adjustment for sex, parents’ level of education and teacher’s assessment were used to estimate the associations between the study variables and attending a higher or lower level of secondary education than indicated by the Cito-test (compared to school level in accordance with the Cito-test). For these analyses the health-related characteristics collected at age 14 were used. These analyses were repeated for attending a higher or lower level of secondary education than indicated by the teacher’s assessment (instead of the Cito-test) as dependent variable. Associations were considered statistically significant at p < 0.05 and analyses were conducted in SAS version 9.3. The model parameters were presented as odds ratios (ORs) and 95% confidence intervals (CI). To gain insight into the potential impact of missing data on our results we compared characteristics of children included in the study and children excluded from the study because of missing data. First, we compared the prevalence of baseline characteristics (paternal and maternal education and ethnicity) of our study population with baseline characteristics of the original total PIAMA population. And second, we compared—within the group of children with data at age 14 (n = 2521)—the prevalence of socio-demographic, health and lifestyle characteristics of children with Cito-test scores (n = 1787) and children without Cito-test scores (n = 734).

## Results

### Sample characteristics

Table 1 shows the sample characteristics. The percentage of highly educated parents in the study population was high (43.6% of the mothers and 48.6% of the fathers), as compared to the percentage in the PIAMA baseline population (35.0% of the mothers and 39.7% of the fathers highly educated) and higher than in the general population of the Netherlands in the period of recruitment (28% of men and women combined in 1997) (CBS). The mean score on the Cito-test was 539.2 which is higher than the mean score in the general population in 2008 (535.4) [19]. For 28.2% of the children, the Cito-test corresponded with the highest level of secondary education (compared to 18% in the general population in 2005) [20].

**Table 1:** Sample characteristics.

Characteristics		PIAMA baseline population (n = 3963)	Study sample (n = 1510)
Ethnicity %			
	Dutch	90.3	91.8
	Non-Dutch, Western	3.4	3.4
	Non-Dutch, not Western	6.2	4.7
Sex %			
	Boy	48.2	47.6
	Girl	51.8	52.4
Highest obtained level of education mother* %			
	Low	23.5	16.7
	Medium	41.6	39.7
	High	35.0	43.6
Highest obtained level of education father* %			
	Low	25.9	19.8
	Medium	34.4	31.6
	High	39.7	48.6
Cito-test indication / teacher’s assessment / level of education at age 14%			
	Lower then pre-vocational secondary education		10.3 / 7.8 / 9.0
	Pre-vocational secondary education		7.4 / 10.2 / 19.3
	Pre-vocational secondary education/senior general education		14.1 / 13.2 / 0.2
	Senior general education		18.1 / 16.9 / 30.9
	Senior general education/pre-university education		22.1 / 21.3 / 1.8
	Pre-university education		28.2 / 30.6 / 38.8

* high: higher vocational education or university; medium: intermediate vocational education or intermediate/higher secondary education; low: primary school, lower vocational or lower secondary education.

### Characteristics associated with the discrepancy between children’s potential and attained level of education

At the age of 14 years, 14.5% of the children attended a higher level of secondary education than indicated by the Cito-test and 13.6% a lower level. S2 Table shows all variables that were studied in relation to discrepancies between Cito-test scores and secondary school level by the use of simple regression analyses. Among the children who received a teacher’s assessment below the level indicated by the Cito-test (n = 93; 5.7% of all children), 56.6% actually attended a lower level of secondary education than their Cito-test indicated. Furthermore, attending a level of secondary education below the level indicated by the Cito-test was significantly more frequent among children with the following characteristics: being a boy, low parental education, having an attention disorder, use of alcohol, drugs and smoking, skipping lessons, being bullied, divorce of parents and nasty experiences. The percentage of children attending a school level below expectations was 16.4% among children who had been ill for more than 3 days in the last 2 months as compared to 13.7% among children who had not been ill, but this difference was not statistically significant. None of the other indicators of physical health were statistically significant associated with attending a lower level of education than expected. Among the children who received a teacher’s assessment above the level indicated by the Cito-test (n = 140; 8.5% of all children), 67.2% actually attended a higher level of secondary education than their Cito-test indicated. Apart from that, only few other factors were statistically significant associated with a higher percentage of children attending a higher level of secondary education than the Cito-test indicated: having a learning disability (like dyslexia or dyscalculia), going to bed early and being a morning person.

Table 2 shows the crude and adjusted associations between the statistically significant factors (based on simple regression analyses as presented in S2 Table) and attending a *lower* level of secondary education than expected based on the Cito-test. The adjusted analyses show that boys were statistically significant more likely than girls to attend a lower level of education than expected and this was also the case for children of fathers with a low level of education. For low maternal education the association was weaker and not statistically significant. The health-related behaviors smoking, use of alcohol and use of drugs were statistically significant and strongly associated with increased risk to attend a lower level of education than expected, with odds ratios ranging from 2.2 (CI: 1.5–3.3) to 5.0 (CI: 2.8–8.7). Furthermore, skipping lessons, attention disorders, divorced parents and nasty experiences were statistically significant associated with increased risk to attend a lower level of education than expected. The association with being bullied lost statistical significance in the adjusted analyses.

**Table 2:** Associations between child characteristics and lower secondary school level than indicated by Cito-test.

Characteristics		School level < Cito-test score (ref. school level = Cito-test score)			
		Crude OR (95% CI)		Adjusted OR* (95% CI)	
Teacher’s assessment (ref. teacher’s assessment = Cito-test score)					
	< Cito-test score	**8.6**	**(5.2 to 14.1)**	**7.6**	**(4.5 to 12.7)**
Male sex		**1.8**	**(1.3 to 2.4)**	**1.9**	**(1.4 to 2.6)**
Education mother (ref. high)					
	Low	**2.4**	**(1.6 to 3.6)**	1.4	(0.8 to 2.3)
	Medium	**1.8**	**(1.3 to 2.6)**	1.4	(1.0 to 2.1)
Education father (ref. high)					
	Low	**2.8**	**(2.0 to 4.1)**	**2.1**	**(1.3 to 3.4)**
	Medium	**1.6**	**(1.1 to 2.3)**	1.3	(0.9 to 2.0)
Diagnosed attention disorder		**3.2**	**(1.9 to 5.4)**	**2.5**	**(1.4 to 4.5)**
Asthma		**0.5**	**(0.2 to 1.0)**	0.5	(0.2 to 1.1)
Smoking (ref. never smoked)					
	Now and then or daily	**2.7**	**(2.0 to 3.7)**	**3.1**	**(2.2 to 4.3)**
Using drugs (ref. never used drugs)					
	Have used or using drugs	**4.1**	**(2.4 to 6.9)**	**5.0**	**(2.8 to 8.7)**
Glasses of alcohol in the past four weeks (ref. no alcohol used)					
	≥ 1 glasses	**1.9**	**(1.4 to 2.8)**	**2.2**	**(1.5 to 3.3)**
Lessons skipped during past four weeks (ref. 0)					
	≥ 1 Lessons	**1.7**	**(1.1 to 2.7)**	**1.7**	**(1.1 to 2.8)**
Parents divorced		**1.5**	**(1.0 to 2.2)**	**1.6**	**(1.0 to 2.4)**
Nasty experiences (ref. few)					
	Many nasty experiences	**1.7**	**(1.2 to 2.6)**	**2.0**	**(1.3 to 3.1)**
Being bullied during the last 12 months (ref. not bullied)					
	Being bullied ≥ 1 time	**1.6**	**(1.1 to 2.3)**	1.4	(0.9 to 2.1)

Statistically significant associations are printed in bold.

* Adjusted for teacher’s assessment < Cito-test score, sex, level of education mother and level of education father.

Table 3 shows the associations between the statistically significant factors (based on bivariate analyses as presented in S2 Table) and attending a *higher* level of secondary education than expected based on the Cito-test. The adjusted analyses indicated that children who were diagnosed with a learning disability and children who went to bed at 10:00 pm or earlier were significantly more likely to attend a higher level of secondary education than expected. The association with being a morning person was no longer statistically significant in the adjusted analyses. The analyses described in this section were repeated for discrepancies between teacher’s assessment (instead of Cito-test scores) and the level of secondary education actually attended 3 years later and showed very similar results (data available on request).

**Table 3:** Associations between child characteristics and higher secondary school level than indicated by Cito-test.

Characteristics		School level > Cito-test score (ref. school level = Cito-test score)			
		Crude OR (95% CI)		Adjusted OR* (95% CI)	
Teacher’s assessment (ref. teacher’s assessment = Cito-test score)					
	> Cito-test score	**22.1**	**(14.4 to 33.9)**	**24.5**	**(15.6 to 38.4)**
Male sex		**0.8**	**(0.6 to 1.1)**	0.8	(0.6 to 1.1)
Education mother (ref. high)					
	Low	**1.5**	**(1.0 to 2.3)**	1.1	(0.6 to 2.0)
	Medium	**0.8**	**(0.5 to 1.1)**	0.7	(0.4 to 1.0)
Education father (ref. high)					
	Low	**1.2**	**(0.8 to 1.8)**	0.8	(0.4 to 1.4)
	Medium	**0.8**	**(0.6 to 1.2)**	0.7	(0.4 to 1.0)
Diagnosed learning disability		**1.9**	**(1.2 to 2.9)**	**1.7**	**(1.0 to 2.9)**
Bedtimes on schooldays (ref. later than 10:00 pm)					
	10:00 pm or earlier	**1.6**	**(1.1 to 2.3)**	**1.6**	**(1.0 to 2.5)**
Morning or evening person (ref. not a morning person)					
	Morning person	**1.6**	**(1.1 to 2.4)**	1.5	(0.9 to 2.4)

Statistically significant associations are printed in bold.

* Adjusted for teacher’s assessment > Cito-test score, sex, level of education mother and level of education father.

We compared the prevalence of factors associated with educational achievement between children with Cito-test score (n = 1787) and children without Cito-test score (n = 734) (see S3 Table) and observed no relevant differences between the two groups.

## Discussion

The only factors significantly associated with attending a *higher* level of secondary education than indicated by the Cito-test score, were having a learning disability and going to bed at or before 10:00 pm on school days. In contrast, attending a *lower* level of secondary education than indicated by the Cito-test score was associated with a variety of factors: being a boy, low paternal education, having an attention disorder, use of alcohol, drugs and smoking, skipping lessons, having divorced parents and nasty experiences. None of the indicators of physical health included in the study (general health, number of illness-days in the last 2 months, asthma, regular headaches or migraine, and fatigue) were associated with discrepancies between the Cito-test score and the level of secondary education actually attended 3 years later. The large majority of children have no severe health problems and apparently they are able to cope well enough with their physical health problems they do have to achieve school results in line with their estimated potential. Thus, attention disorders and stressful life events seem more likely to affect school careers than physical health. In order to promote equal opportunities for children to achieve their educational potential, it is important that schools support children with attention disorders or children that cope with stressful life events.

Furthermore, use of alcohol and drugs and smoking were strongly associated with attending a lower level of secondary education than expected. These associations may be bi-directional: substance use is likely to have a negative impact on school results, but poor school results may also make children look for satisfaction elsewhere. Although causality cannot be established, our results suggest that smoking and use of alcohol and drugs in adolescence may jeopardize future health in two ways: by direct physiological health effects and by the negative impact they may have on educational attainment, which in turn, is a strong determinant of healthy life expectancy.

We found that when a child was at a higher or lower educational level at age 14 than indicated by the Cito-test score, the teacher’s assessment–if it indicated a different level than the Cito-test score–was a very strong determinant of this discrepancy. The associations between child characteristics and discrepancy between educational potential indicated by the Cito-test score and the attained level of secondary education were assessed in adjusted analyses taking the teacher’s assessment into account. The observed associations with child characteristics are therefore independent of the influence of the teacher’s assessment.

The factors that were associated in our study with attending a lower level of secondary education than expected based on the primary school’s assessment have also been identified in some previous studies as adverse determinants of educational achievements [5,6,9,21–23]. Some, but not all, earlier studies also found associations between educational achievement and TV watching and computer use [24], sleep related factors [7] and being bullied [8,9,25], whereas these factors were not significantly associated with discrepancies between expected and attained level of education in our study.

A recent Dutch prospective study based on educational and medical registries revealed that adolescents who had more frequent contact with their general practitioner for acute psychosocial problems (including for example bedwetting and hyperactivity) were less likely to complete their secondary education and were less likely to complete their secondary education at the level of entry [26]. In our study, we found associations of attention disorders and stressful events (nasty experiences and parental divorce) with lower educational achievement. Although the registry based study [26], covered a broader range of physical health problems (acute somatic health problems, infections, acute somatic traumata and chronic diseases) than the present study, no associations between physical health on school careers were found. In contrast, a number of other studies found that poorer health is associated with poorer school performance of children [9,10,27–29]. However, these studies did not examine the effects of health on discrepancies between expected and attained level of education, as was done in our study. As such, our study adds evidence to the existing literature by providing insights from this specific perspective.

### Strengths and limitations

Strengths of the present study are the longitudinal design and the large range of different child characteristics that could be studied. The study is however also subject to some limitations. Firstly, missing data: a total number of 1510 14-year-olds were included in this study out of the total number of 3963 children included in the PIAMA cohort at birth. For the other children data that were essential for this study were missing, which was caused by two main factors. The first cause is loss-to-follow-up and non-response on specific questionnaires that occurs over the years in all cohort studies. The second cause, that is specific to this particular study, is that–apart from missing questionnaires–Cito-test scores were missing for a substantial number of children with complete questionnaires, either because the school did not offer the Cito-test or because the child was not in the final grade of primary school when the parents received our questionnaire (see Fig 1). We found that children with Cito-test scores and children without Cito-test scores were very similar with respect to the health and lifestyle factors that were associated with educational achievement. There seems to be no reason therefore to assume that the health and lifestyle factors that are associated with educational achievement in our study would not be relevant for the children who were excluded from the study due to missing data on the Cito-test.

Secondly, highly educated parents were overrepresented in the study population. The relatively large proportion of highly educated parents may also explain that a relatively large proportion of the children in our sample obtained Cito-test scores qualifying for the highest level of secondary education (28% in the study population versus 18% in the total Dutch population). These children could attain a lower, but not a higher level of secondary education than expected, which might imply that we underestimated the percentage of children who attend a higher level of education than expected. Similarly, we may have overestimated the percentage of children who attend a lower level of education than expected. However, the percentages of attending higher and lower levels than expected that were observed in our study were very similar to the percentages obtained in the recent study conducted in the Netherlands (15.1% of the children at the beginning of their fourth year in secondary school attended a lower level of education and 14.3% a higher level of education than indicated by the Cito-test results) [12], suggesting that there is no major bias in our study results. Our analyses were adjusted for parental education and we assume that the associations we observed in our sample would also be present in a sample with a more representative distribution of parental education.

Thirdly, all data used in this study were self-reported, either by the parents or the children and may therefore have been influenced by reporting error. It seems unlikely that the associations we observed resulted from reporting error, but we may have ‘missed’ existing associations due to inaccurate reporting. On the other hand, self-reported health outcomes, such as the presence of migraine or stress, can be considered very valuable, as they reflect the participants’ own subjective perception of these complaints. Fourthly, our study was an observational study and causality cannot be established based on our findings. Finally, we did not have any information on school characteristics that may have affected children’s school performance.

## Conclusions

In conclusion, our results suggest that children are able to cope well enough with common physical health problems to avoid a negative impact of illness on their attained level of secondary education compared to their potential as assessed at the end of primary school. In contrast, attention disorders, stressful life events and especially substance use do seem to increase the risk of educational achievement below their potential.

## Supporting Information

S1 Table Classification secondary school levels according to Cito-test scores and school level. (DOCX)

S2 Table Characteristics of children attaining a higher, lower or corresponding secondary school level indicated by Cito-test score (n = 1510). (DOCX)

S3 Table Characteristics of children without a Cito-test score (missing values) compared to children with a Cito-test score (study sample). (DOC)
