# Supplementary material for: Health-Related Factors Associated with Discrepancies between Children’s Potential and Attained Secondary School Level: A Longitudinal Study
Source: PLoS One. 2016 Dec 22;11(12):e0168110. doi: 10.1371/journal.pone.0168110 (PMC5179065; doi:10.1371/journal.pone.0168110)
Supplement: S1 Table — (DOCX) [file pone.0168110.s001.docx]

**S1 Table. Classification secondary school levels according to Cito-test scores and school level.**

| Cito-test score | Dutch school levels | ISCED^a^ | Classification^b^ |
| --- | --- | --- | --- |
| < 523 | Vmbo_b/l | ISCED 2 | 1 |
| ≥ 523 and < 529 | Vmbo_k | ISCED 2 | 2 |
| ≥ 529 and < 533 | Vmbo_g/t | ISCED 2 / pre-vocational secondary education, qualifying for senior secondary vocational education | 3 |
| ≥ 533 and < 537 | Vmbo/Havo | ISCED 2 / 3 / Combination class of pre-vocational secondary education and senior general education | 3.5 |
| ≥ 537 and < 541 | Havo | ISCED 3 / Senior general education, qualifying for higher education | 4 |
| ≥ 541 and < 545 | Havo/Vwo  (only offered in the first year of secondary education) | ISCED 3 / Combination class of senior general education and pre-university education | 4.5 |
| > 545 | Vwo | ISCED 3 / Pre-university education, qualifying for higher education | 5 |

^a^ International Standard Classification of Education by UNESCO (update 1997).

^b^ Adapted version of the classification proposed by Bosker and colleagues (1985); half point score is possible based on advice or cito-test score, but three years later the categories of vmbo/havo (3.5) and havo/vwo (4.5) are no longer available because this is only offered in the first year of secondary education.
