# Supplementary material for: Health-Related Factors Associated with Discrepancies between Children’s Potential and Attained Secondary School Level: A Longitudinal Study
Source: PLoS One. 2016 Dec 22;11(12):e0168110. doi: 10.1371/journal.pone.0168110 (PMC5179065; doi:10.1371/journal.pone.0168110)
Supplement: S2 Table — (DOCX) [file pone.0168110.s002.docx]

**S2 Table. Characteristics of children attaining a higher, lower or corresponding secondary school level indicated by Cito-test score (n=1510).**

| Characteristics | | | Categories for Cito-test scores versus  attained level of secondary education | | |
| --- | --- | --- | --- | --- | --- |
|  | | | School level =  Cito-test (n=1085) | School level <  Cito-test (n=219) | School level >  Cito-test (n=206) |
| Characteristics before age 14 | | |  |  |  |
| *Socio-demographic characteristics* | |  |  |  |  |
| Ethnicity child % | |  |  |  |  |
|  | | Dutch (n=1362)^*^ | 71.2 | 4.8 | 14.1 |
|  | | Non-Dutch, Western (n=51) | 78.4 | 9.8 | 11.8 |
|  | | Non-Dutch, not Western (n=70) | 75.7 | 15.7 | 8.6 |
| Sex % | |  |  |  |  |
|  | | Boy (n=719) | 69.7 | 18.4 | 12.0 |
|  | | Girl (n=791) | 73.8 | 11.0 | 15.2 |
| Highest obtained level of education mother ^a^  % | |  |  |  |  |
|  | | High (n=658)^*^ | 75.5 | 9.9 | 14.6 |
|  | | Medium (n=599) | 72.0 | 17.4 | 10.7 |
|  | | Low (n=252) | 62.3 | 19.4 | 18.3 |
| Highest obtained level of education father ^a^ % | |  |  |  |  |
|  | | High (n=727)^*^ | 75.4 | 10.0 | 14.6 |
|  | | Medium (n=473) | 72.7 | 15.4 | 11.8 |
|  | | Low (n=296) | 61.8 | 23.3 | 14.9 |
| *Teacher’s assessment versus Cito-test* | |  |  |  |  |
| Teacher’s assessment versus Cito-test % | |  |  |  |  |
|  | | Assessment = Cito-test (n=1285)^*^ | 78.5 | 13.1 | 8.4 |
|  | | Assessment < Cito-test (n=76) | 39.5 | 56.6 | 4.0 |
|  | | Assessment > Cito-test (n=134) | 28.4 | 4.5 | 67.2 |
| Characteristics at age 14 | | |  |  |  |
| *Health* |  | |  |  |  |
| Perceived health according to child % |  | |  |  |  |
|  | Good/excellent (n=1454) | | 71.5 | 14.5 | 14.0 |
|  | Moderate/bad (n=56) | | 80.4 | 14.3 | 5.4 |
| Number of days ill at home during last 2 months % |  | |  |  |  |
|  | 0 (n=832)^*^ | | 71.3 | 13.7 | 15.0 |
|  | 1 – 2 (n=420) | | 72.9 | 15.2 | 11.9 |
|  | > 3 (n=171) | | 71.4 | 16.4 | 12.3 |
| Diagnosed learning disability like dyslexia or dyscalculia % |  | |  |  |  |
|  | No (n=1257) | | 73.0 | 14.2 | 12.7 |
|  | Yes (n=162) | | 62.4 | 17.3 | 20.4 |
| Diagnosed attention disorder like ADD  or ADHD % |  | |  |  |  |
|  | No (n=1337) | | 73.0 | 13.5 | 13.5 |
|  | Yes (n=80) | | 52.5 | 31.3 | 16.3 |
| Diagnosed autistic disorder like PDD-NOS or Asperger % |  | |  |  |  |
|  | No (n=1362) | | 72.2 | 14.3 | 13.5 |
|  | Yes (n=54) | | 64.8 | 18.5 | 16.7 |
| Asthma % |  | |  |  |  |
|  | No (n=1400) | | 71.6 | 15.1 | 13.3 |
|  | Yes (n=109) | | 74.3 | 7.3 | 18.4 |
| Migraine % |  | |  |  |  |
|  | No (n=1352) | | 71.5 | 14.7 | 13.8 |
|  | Yes (n=157) | | 75.2 | 12.7 | 12.1 |
| Fatigue % |  | |  |  |  |
|  | No (n=1341) | | 71.4 | 14.4 | 14.2 |
|  | Yes (n=168) | | 75.6 | 15.5 | 8.9 |
| Mental health ^b^ % |  | |  |  |  |
|  | Good mental health (n=1337) | | 72.3 | 14.5 | 13.2 |
|  | Poor mental health (n=171) | | 67.8 | 14.6 | 17.5 |
| *Sleeping behavior* |  | |  |  |  |
| Wake up in the night % |  | |  |  |  |
|  | Not often/not for long (n=1202)^*^ | | 72.1 | 14.6 | 13.4 |
|  | Occasionally/for some time (n=238) | | 70.2 | 14.7 | 15.1 |
|  | Often/for a long time (n=67) | | 73.1 | 13.4 | 13.4 |
| Difficulty getting up in the morning % |  | |  |  |  |
|  | Little difficulty getting (n=987)^*^ | | 71.5 | 14.7 | 13.8 |
|  | Difficulty getting up (n=521) | | 72.4 | 14.2 | 13.4 |
| Feeling rested after waking up on school days % |  | |  |  |  |
|  | Feeling rested (n=866)^*^ | | 73.2 | 13.6 | 13.2 |
|  | Not feeling rested (n=641) | | 70.1 | 15.8 | 14.2 |
| Bed times on school days % |  | |  |  |  |
|  | 10:00 pm or earlier (n=1102) | | 71.4 | 13.4 | 15.2 |
|  | Later than 10:00 pm (n=405) | | 72.8 | 17.5 | 9.6 |
| Morning person or evening person % |  | |  |  |  |
|  | Morning person (n=190) | | 70.0 | 10.0 | 20.0 |
|  | Not explicit morning or evening person (n=617)^*^ | | 73.0 | 14.6 | 12.3 |
|  | Evening person (n=694) | | 71.5 | 15.7 | 12.8 |
| *Lifestyle* |  | |  |  |  |
| Hours per week spent watching TV or using computer % |  | |  |  |  |
|  | ≤ 18 hours (n=810) | | 73.2 | 13.0 | 13.8 |
|  | > 18 hours (n=700) | | 70.3 | 16.3 | 13.4 |
| Glasses of alcohol in the past four weeks % |  | |  |  |  |
|  | 0 (n=1258) | | 73.4 | 13.0 | 13.6 |
|  | ≥ 1 glasses (n=249) | | 63.9 | 22.1 | 14.1 |
| Using drugs % |  | |  |  |  |
|  | Never (n=1442) | | 72.6 | 13.3 | 14.1 |
|  | Ever (n=66) | | 54.6 | 40.9 | 4.6 |
| Smoking % |  | |  |  |  |
|  | Never (n=1145) | | 74.8 | 11.1 | 14.2 |
|  | Ever (n=361) | | 62.6 | 25.5 | 11.9 |
| Number of lessons skipped during the past 4 weeks % |  | |  |  |  |
|  | 0 (n=1307) | | 73.0 | 13.3 | 13.7 |
|  | ≥1 (n=135) | | 68.2 | 21.5 | 10.4 |
| *Stressful life events* |  | |  |  |  |
| Problems that keep you busy day and night % |  | |  |  |  |
|  | No (n=1329) | | 71.7 | 14.6 | 13.7 |
|  | Yes (n=178) | | 72.5 | 14.0 | 13.5 |
| Death of a family member or other close person % |  | |  |  |  |
|  | No (n= 797) | | 73.0 | 13.2 | 13.8 |
|  | Yes (n= 629) | | 70.1 | 16.1 | 13.8 |
| Parents divorced % |  | |  |  |  |
|  | No (n=1226) | | 72.5 | 13.7 | 13.8 |
|  | Yes (n=200) | | 67.0 | 19.0 | 14.0 |
| Being bullied during the last 12 months % |  | |  |  |  |
|  | Never (n=1296) | | 73.0 | 13.7 | 13.3 |
|  | ≥ once (n=214) | | 65.0 | 19.2 | 15.9 |
| Nasty experiences ^c^ % |  | |  |  |  |
|  | Few (n=1320) | | 73.0 | 13.6 | 13.3 |
|  | Many (n=190) | | 63.7 | 20.5 | 15.8 |

Significant differences based on bivariate analyses are printed in bold.

^*^ Reference category.

^a^ high: higher vocational education or university; medium: intermediate vocational education or intermediate/higher secondary education; low: primary school, lower vocational or lower secondary education.

^b^ Good mental health: MHI5 > 60; poor mental health: MHI5 < 61.

^c^ Few nasty experiences (scores 0-5) and many nasty experiences (scores 6-10).
