# Supplementary material for: Health-Related Factors Associated with Discrepancies between Children’s Potential and Attained Secondary School Level: A Longitudinal Study
Source: PLoS One. 2016 Dec 22;11(12):e0168110. doi: 10.1371/journal.pone.0168110 (PMC5179065; doi:10.1371/journal.pone.0168110)
Supplement: S3 Table — (DOC) [file pone.0168110.s003.doc]

**S3 Table. Characteristics of children without a Cito-test score (missing values) compared to children with a Cito-test score (study sample).**

| Characteristics |  | Children without a Cito-test score (n=734) % (n) | Children with a Cito-test score (n=1787) % (n) |
| --- | --- | --- | --- |
| Sex |  |  |  |
|  | Boy | 49.7 (365) | 50.5 (902) |
|  | Girl | 50.3 (369) | 49.5 (884) |
| Highest obtained level of education mother ^*^ |  |  |  |
|  | Low | 20.2 (148) | 18.7 (334) |
|  | Medium | 41.0 (301) | 40.3 (721) |
|  | High | 38.4 (282) | 40.7 (727) |
| Highest obtained level of education father ^*^ |  |  |  |
|  | Low | 21.4 (157) | 22.2 (397) |
|  | Medium | 33.8 (248) | 33.1 (591) |
|  | High | 43.5 (319) | 43.7 (781) |
| Diagnosed learning disability like dyslexia or dyscalculia |  | 14.2 (104) | 13.6 (243) |
| Parents divorced |  | 14.9 (109) | 14.7 (262) |
| Asthma |  | 91.7 (673) | 92.6 (1654) |
| Being bullied during the last 12 months |  |  |  |
|  | Never | 82.2 (603) | 85.1 (1520) |
|  | ≥ once | 17.6 (129) | 14.5 (260) |
| Nasty experiences |  |  |  |
|  | Few | 88.0 (646) | 86.1 (1539) |
|  | Many | 11.7 (86) | 13.4 (240) |
| Number of lessons skipped during the past 4 weeks |  |  |  |
|  | 0 | 85.6 (628) | 84.2 (1505) |
|  | ≥1 | 9.8 (72) | 8.9 (160) |
| Glasses of alcohol in the past four weeks |  |  |  |
|  | 0 | 33.9 (249) | 34.8 (622) |
|  | ≥ 1 | 36.1 (265) | 35.3 (631) |
| Smoking |  |  |  |
|  | Never | 72.5 (532) | 75.7 (1353) |
|  | Ever | 26.8 (197) | 23.4 (419) |
| Using drugs |  |  |  |
|  | Never | 93.7 (688) | 94.3 (1686) |
|  | Ever | 5.7 (42) | 4.9 (87) |
| Bed times on school days |  |  |  |
|  | 10:00 pm or earlier | 72.5 (532) | 75.2 (1343) |
|  | Later than 10:00 pm | 27.2 (200) | 24.1 (430) |
| Morning person or evening person |  |  |  |
|  | Morning person | 13.2 (97) | 11.8 (211) |
|  | Not explicit morning or evening person | 38.7 (284) | 41.7 (746) |
|  | Evening person | 47.4 (348) | 45.3 (809) |

^*^ high: higher vocational education or university; medium: intermediate vocational education or intermediate/higher secondary education; low: primary school, lower vocational or lower secondary education.
